# PhasiHunter: a robust phased siRNA regulatory cascade mining tool based on multiple reference sequences

**DOI:** 10.1093/bioinformatics/btad676

**Published:** 2023-11-09

**Authors:** Zerong Feng, Jiejie Feng, Baoyi Zhang, Yuhan Fei, Hongsheng Zhang, Ji Huang

**Affiliations:** State Key Laboratory of Crop Genetics & Germplasm Enhancement and Utilization, Jiangsu Province Engineering Research Center of Seed Industry Science and Technology, Nanjing Agricultural University, Nanjing 210095, China; State Key Laboratory of Crop Genetics & Germplasm Enhancement and Utilization, Jiangsu Province Engineering Research Center of Seed Industry Science and Technology, Nanjing Agricultural University, Nanjing 210095, China; State Key Laboratory of Crop Genetics & Germplasm Enhancement and Utilization, Jiangsu Province Engineering Research Center of Seed Industry Science and Technology, Nanjing Agricultural University, Nanjing 210095, China; MOE Key Laboratory of Bioinformatics, Beijing Advanced Innovation Center for Structural Biology & Frontier Research Center for Biological Structure, Center for Synthetic and Systems Biology, School of Life Sciences, Tsinghua University, Beijing 100084, China; State Key Laboratory of Crop Genetics & Germplasm Enhancement and Utilization, Jiangsu Province Engineering Research Center of Seed Industry Science and Technology, Nanjing Agricultural University, Nanjing 210095, China; State Key Laboratory of Crop Genetics & Germplasm Enhancement and Utilization, Jiangsu Province Engineering Research Center of Seed Industry Science and Technology, Nanjing Agricultural University, Nanjing 210095, China; Jiangsu Key Laboratory for Information Agriculture, Nanjing Agricultural University, Nanjing 210095, China

## Abstract

**Summary:**

In recent years, phased small interfering RNA has been found to play crucial roles in many biological processes in plants. However, efficiently predicting phasiRNA regulatory cascades with computational methods is still challenging. Here, we introduce PhasiHunter, a phasiRNA regulatory network prediction tool that has several distinctive features compared to existing tools: (i) PhasiHunter employs two major phasiRNA prediction algorithms, namely phase score and hypergeometric distribution-based methods, to ensure the integrity and accuracy of prediction; (ii) PhasiHunter can identify phasiRNAs and their regulatory networks based on multiple reference sequences and the predicted results can be automatically integrated; (iii) PhasiHunter can efficiently identify the phasiRNAs generated through alternative splicing events; and (iv) the excellent data structure and parallel computing architecture allow PhasiHunter to predict phasiRNAs and their regulatory pathways with high efficiency.

**Availability and implementation:**

PhasiHunter is an open-source tool that is available at https://github.com/HuangLab-CBI/PhasiHunter.

## 1 Introduction

PhasiRNAs are a type of small RNA in plants that are generated from mRNA or ncRNA directly with high abundance and play an important role in abiotic stress resistance, plant immunity and plant development (Deng *et al.* 2018, Liu *et al.* 2020, Zhan and Meyers 2023).

The prediction of phasiRNAs based on transcriptome is more suitable in biology meaning than genome; however, the incomplete annotation of transcriptome is limited in the application of phasiRNA prediction. Some of the flaws can be overcome by prediction based on genome and transcriptome simultaneously. Several bioinformatics tools such as PhaseTank (Guo *et al.* 2015), unitas (Gebert *et al.* 2017) and PhasiRNAnalyzer (Fei *et al.* 2021) have been developed to predict phasiRNAs in plants. However, no tools could predict phasiRNAs based on multiple reference sequences, integrate the prediction results and annotate phasiRNA regulatory cascades in detail.

In this study, we introduce a novel multithreading tool PhasiHunter to identify phasiRNA regulatory cascades, which can comprehensively utilize multiple reference sequences (genome, transcriptome and full-length transcriptome) information for predicting phasiRNAs and their regulatory networks. Additionally, PhasiHunter combines the *P*-value-based method (Chen *et al.* 2007) and the phase score-based method (Axtell 2010, Guo *et al.* 2015) for more sensitive and accurate phasiRNA prediction.

## 2 Approach

### 2.1 PhasiRNA prediction based on multiple reference sequences

The prediction of phasiRNA based on multiple reference sequences is achieved in two steps. Firstly, PhasiHunter executes phasiRNA prediction based on different single reference sequences simultaneously with multiple threads, and then PhasiHunter integrates the prediction results based on different reference sequences by the coordinate system transform which is achieved by the bedtools (Quinlan and Hall 2010) and the dictionary data structure in python3.

### 2.2 PhasiRNA cluster prediction algorithm

Phase score (Axtell 2010, Guo *et al.* 2015) and hypergeometric distribution-based methods (Chen *et al.* 2007) are two popular algorithms for predicting *PHAS* loci. PhasiHunter integrates two algorithms for the prediction of *PHAS* loci for acquiring more accurate and comprehensive results (Supplementary Fig. S1A). In the hypergeometric distribution algorithm, we set the forward and reverse search functions for enhancing the *PHAS* loci detection sensitivity (Supplementary Fig. S1B).

### 2.3 Parallel computing design

To improve the phasiRNA prediction speed, a highly parallel computing function was designed in PhasiHunter (Supplementary Fig. S2). A process pool was created by the python3 standard library “concurrent”, and phasiRNA prediction on different transcripts or chromosomes will be added to the process pool as a child process. Users can control the maximum number of processes to achieve fast phasiRNA prediction.

### 2.4 Prediction and verification of phase initiator and phasiRNA target

PhasiHunter employs the same method as PhasiRNAnalyzer (Fei *et al.* 2021) to predict phase initiator. The identification of interaction between miRNA or phasiRNA and their target genes is achieved by TarHunter (Ma *et al.* 2018) or psRNATarget (Dai *et al.* 2018) algorithm.

## 3 Workflow and implementation

**Figure 1.**
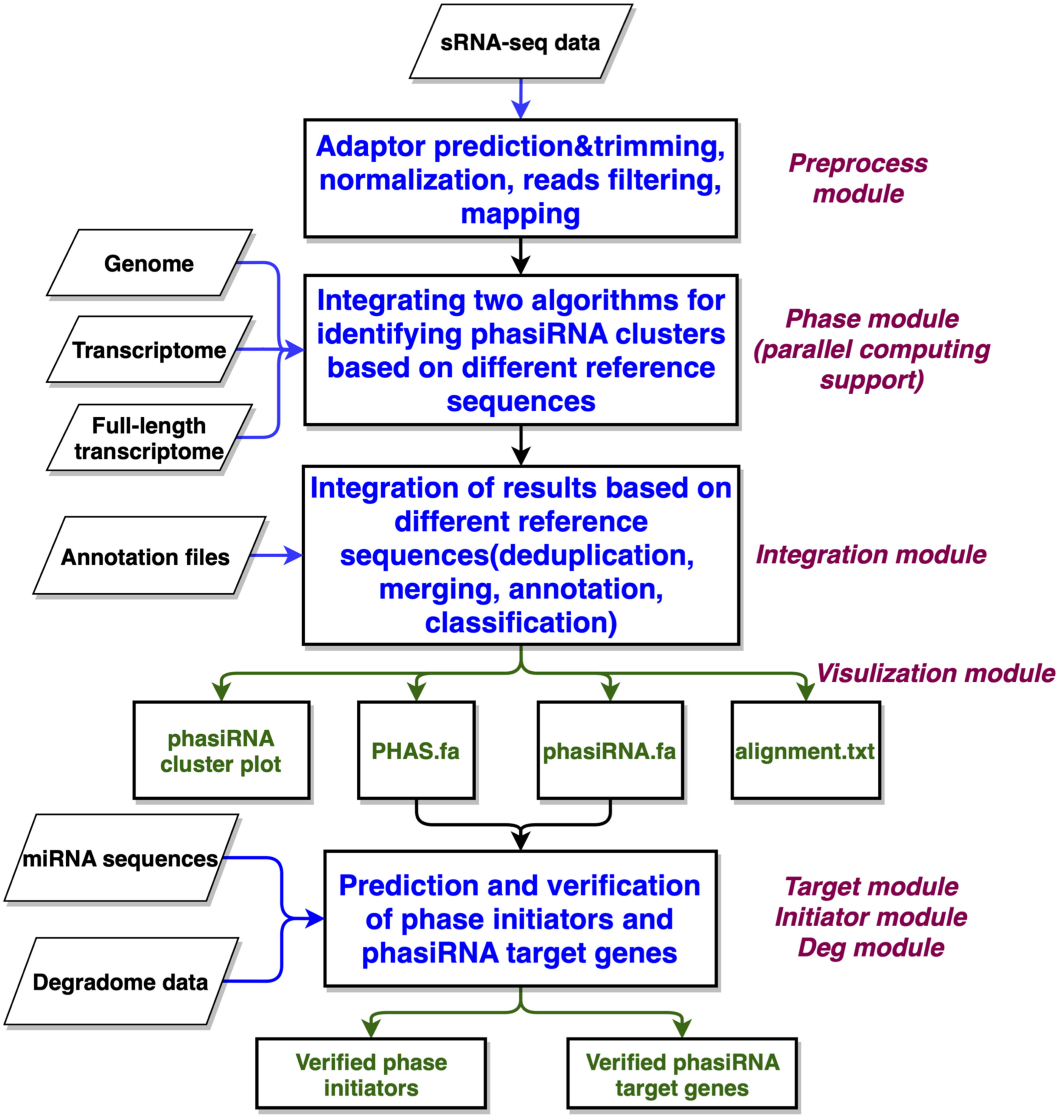
Framework of PhasiHunter.

PhasiHunter contains seven modules for phasiRNA regulatory cascades identification and verification (Fig. 1). The preprocess module is employed to process the FASTQ or FASTA file. In this step, adaptor prediction, adaptor trimming, normalization, reads length filtering, reads expression filtering, and sRNA mapping are executed. The phase module uses the algorithms of hypergeometric distribution and phase score to predict phasiRNAs based on different reference sequences concurrently with multithreading. The deduplication, merging, annotation, and classification of phasiRNA clusters based on different reference sequences are conducted by the integration module based on GFF3 annotation files and full-length transcriptome annotation files. The phasiRNA cluster plot, phasiRNA alignment file, *PHAS* loci sequence file and phasiRNA sequence file are generated by the visualization module. The target module and initiator module can achieve the predictions of phase initiators and phasiRNA target genes. The deg module can verify the phasiRNA-target prediction results based on degradome sequencing data.

PhasiHunter is a command line interface program written in python3 (3.6 or later version) and has been tested in Ubuntu 16.04. Some dependencies are required for properly running PhasiHunter, including bowtie (Langmead *et al.* 2009), dnapi (Tsuji and Weng 2016), seqkit (Shen *et al.* 2016), trim_galore (https://github.com/FelixKrueger/TrimGalore), Biopython (Cock *et al.* 2009), Perl5 (https://www.perl.org) and fasta36 (Pearson and Lipman 1988). To facilitate ease of use, we offer a preconfigured docker image encompassing all the necessary dependencies for running PhasiHunter. Additionally, for conda users, we provide a comprehensive environment configuration file that encompasses all the required dependencies. For further information, detailed instructions can be accessed through the PhasiHunter GitHub repository or user manual.

## 4 Results

With the test dataset (Supplementary Table S1), which is constructed by 32 sRNA-seq libraries from maize (Zhai *et al.* 2015), we compared the phasiRNA prediction results based on different reference sequences (Supplementary Fig. S3). It can be observed that the prediction results based on different reference sequences contain some specific *PHAS* genes. Thirty and 95 unique *PHAS* genes can be found, respectively, when comparing the transcriptome-based and genome-based phasiRNA prediction results. Among the 30 unique *PHAS* genes based on transcriptome, 60% of them have phasiRNA-generating loci located in multi-exon, while the remaining 40% have phasiRNA-generating loci located in mono-exon. Among the 95 unique *PHAS* genes based on genome, 69.5% of them have phasiRNA-generating loci spanning intron and exon, and 1% of them have phasiRNA-generating loci located in intron. A total of 122 unique *PHAS* genes can be found in prediction results based on full-length transcriptome, of which almost all *PHAS* genes (96.7%) are novel unannotated genes or involve novel alternative splicing according to the annotation of SQANTI3 program (Tardaguila *et al.* 2018).

To evaluate the performance of PhasiHunter, we applied PhasiHunter, unitas, PhaseTank, and PhasiRNAnalyzer on the test dataset. The 21-*PHAS* gene prediction results of different softwares based on transcriptome (Supplementary Fig. S4A) showed that PhasiHunter and PhasiRNAnalyzer predicted more *PHAS* genes (181 and 178, respectively), while unitas and PhaseTank predicted fewer *PHAS* genes. Of the results predicted by PhasiRNAnalyzer, 94.4% (168) can be predicted by one or more other software, while 96.7% (175) of the results predicted by PhasiHunter can be also identified by one or more other software. There are some specific *PHAS* genes predicted in each software, and we found that the specific *PHAS* genes in PhasiRNAnalyzer had lower phasiRNAs expression values and their *P*-value was close to the threshold. Although the *P*-value of the specific *PHAS* genes in PhasiHunter was also close to the threshold, but the expression value of phasiRNAs was relatively higher (Supplementary Fig. S4C). PhaseTank-specific *PHAS* genes contain a small number of phase number usually between 4 and 7 (Supplementary Table S2). The length of phasiRNA-generating loci of the only *PHAS* gene specific to unitas is 1111 bp, but the phase number contained in it is only 8 (Supplementary Table S3). PhasiHunter predicted 99 additional specific *PHAS* genes than other software when executing phasiRNA prediction based on genome and transcriptome simultaneously (Supplementary Fig. S4B, Supplementary Table S4). Besides, according to the integration information of phasiRNA prediction results based on transcriptome and genome, the user can identify the phasiRNAs specifically predicted through AS events efficiently. We also presented a case related to AS using IGV-sRNA (https://gitee.com/CJchen/IGV-sRNA) (Supplementary Fig. S5). In this case, LOC103650958 can produce four transcript isoforms whereas one of which is unable to generate phasiRNAs due to intron clipping.

We also compared the time cost of 21-nt phasiRNA prediction based on genome or transcriptome with different software. The execution time for a common step (index preparation and sRNA mapping) performed by bowtie in four software are excluded. In the prediction of phasiRNA based on transcriptome (Supplementary Fig. S6A), the running time of unitas and PhasiHunter in single-threaded mode is close (17.56 and 21.41 min). The running time of PhaseTank is slightly higher (35.24 min) and PhasiRNAnalyzer took the most time (181.93 min, 10 times of unitas time consumption, 8 times of PhasiHunter time consumption). In the prediction of phasiRNA based on genome (Supplementary Fig. S6B), PhasiHunter took the least time at 91.03 min in single-threaded mode, followed by unitas, PhaseTank, and PhasiRNAnalyzer at 158.30, 315.55, and 919.32 min, respectively. In addition, only PhasiHunter provides a parallel computing function, and the running time can be reduced by increasing the number of threads. When predicting based on genome, the time consumption of PhasiHunter in four-thread mode was reduced to about 45 min.

## 5 Conclusion

In conclusion, PhasiHunter is a reliable and multiple threads-based tool for predicting plant phasiRNA regulatory cascades by integrating two prediction algorithms and it supports the automatic integration of the prediction with multiple reference sequences at one time.

## Supplementary Material

btad676_Supplementary_Data

## Data Availability

The data underlying this article are available in the article and in its online supplementary material.
