# Supplementary material for: PhasiHunter: a robust phased siRNA regulatory cascade mining tool based on multiple reference sequences
Source: Bioinformatics. 2023 Nov 9;39(11):btad676. doi: 10.1093/bioinformatics/btad676 (PMC10651429; doi:10.1093/bioinformatics/btad676)
Supplement: btad676_Supplementary_Data [file btad676_supplementary_data.zip › Supplementary_Figures v1.3.pdf]

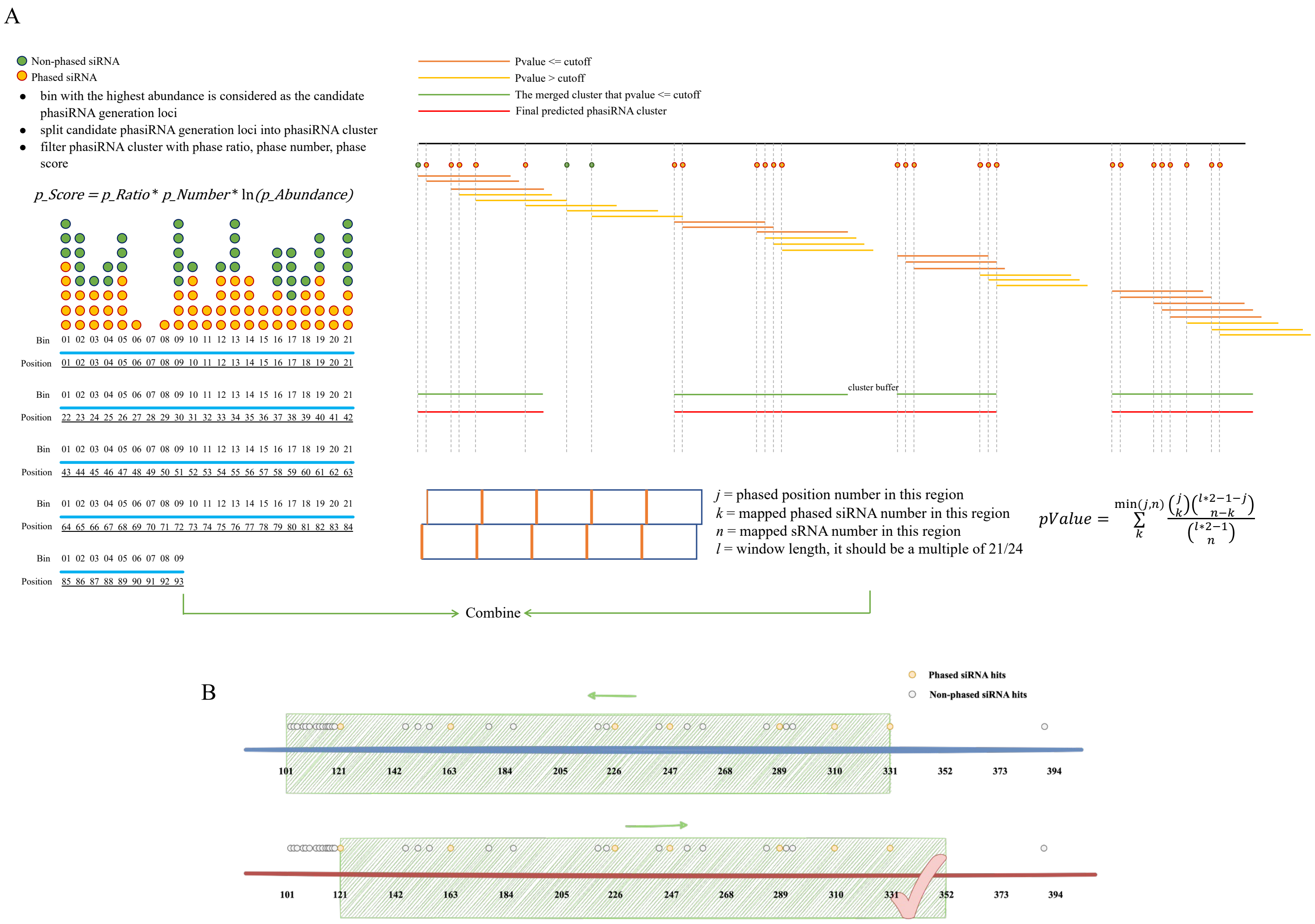

$$p\_Score = p\_Ratio * p\_Number * \ln(p\_Abundance)$$

$$pValue = \sum_{k}^{\min(j,n)} \frac{\binom{j}{k}\binom{l*2-1-j}{n-k}}{\binom{l*2-1}{n}}$$

Figure S1. PhasiRNA cluster prediction algorithm

(A) Illustration of the phasiRNA cluster prediction algorithm. Phase score algorithm is shown on the left, hypergeometric test algorithm is shown on the right.

(B) Forward and backward search scheme for hypergeometric test. The top side shows an example of a reverse search, the bottom side shows an example of a forward search, and the red tick indicates that the p-value in this interval is less than a preset threshold.

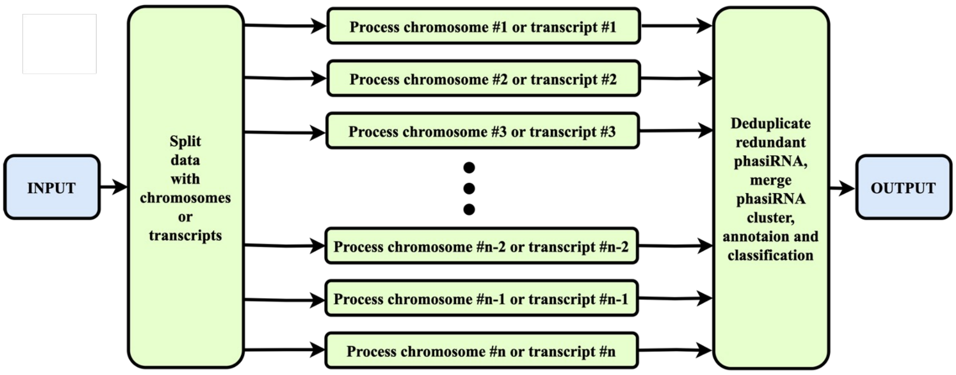

Figure S2. Design of parallel computing in PhasiHunter

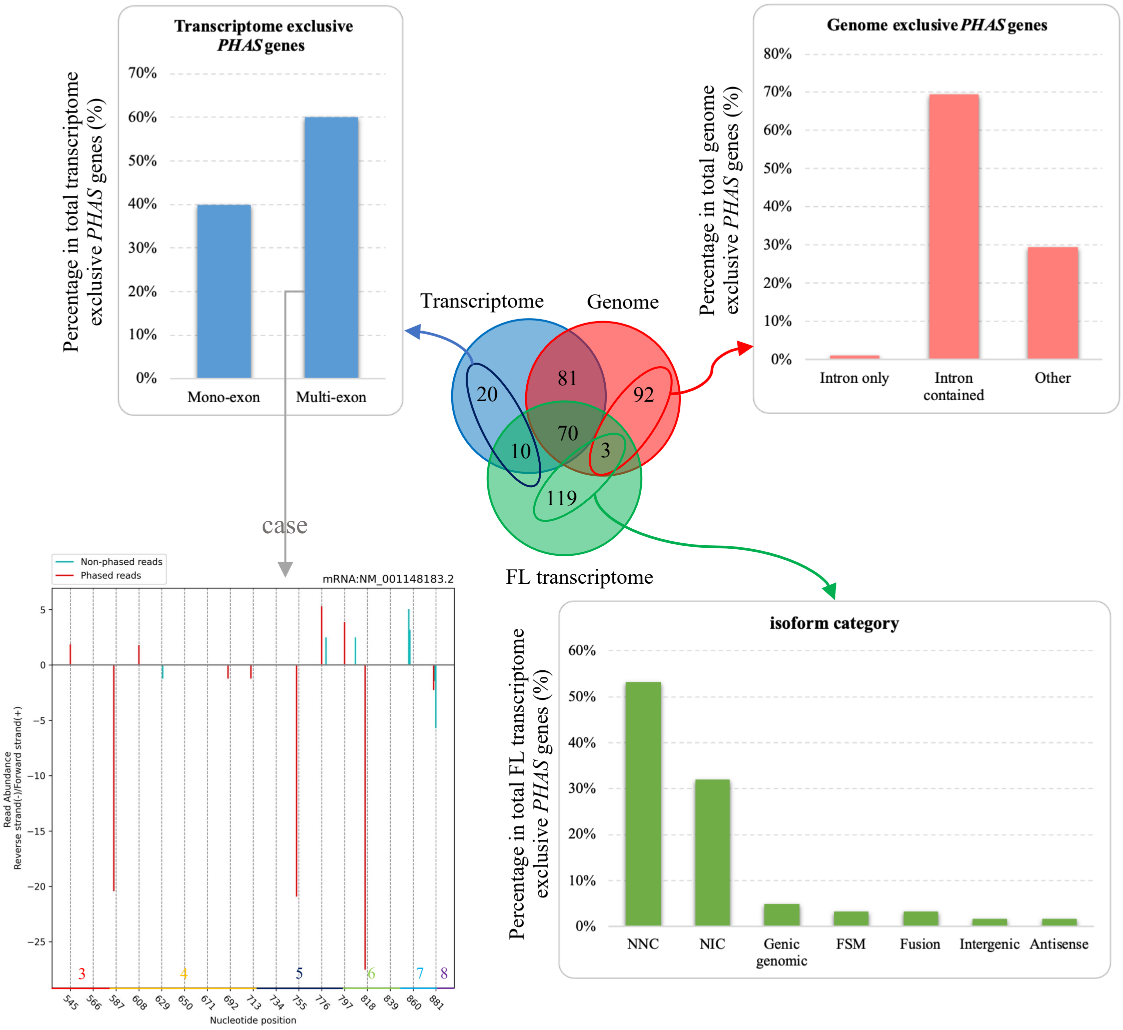

Figure S3. Comparison of predicted *PHAS* genes based on different references.
A phasiRNA cluster plot in the lower leaf presents a case of a *PHAS* gene with phasiRNA-generating loci spanning six exons. Abbreviations: NNC, Novel not in catalog; NIC, Novel in catalog; FSM, Full splice match.

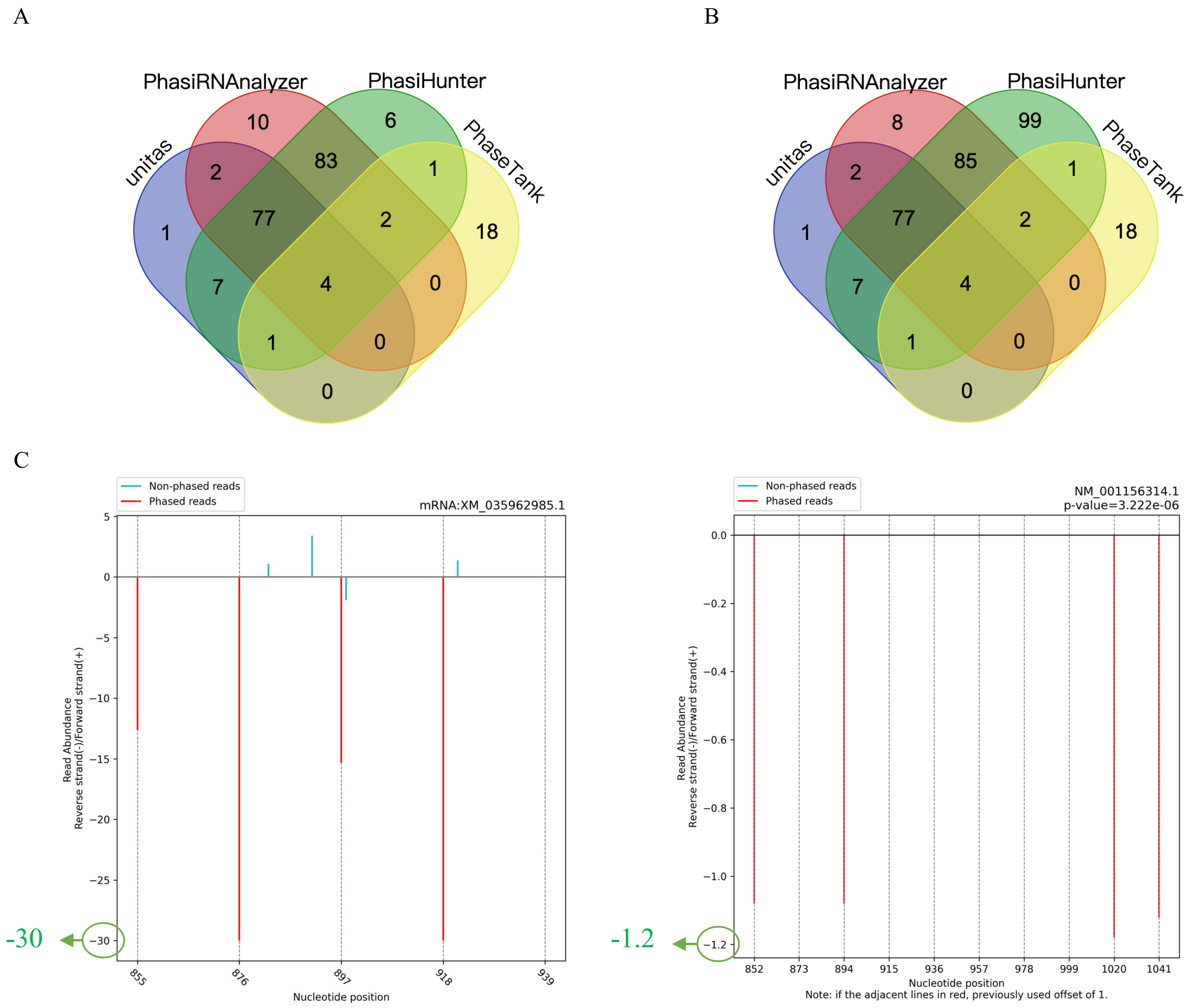

Figure S4. Comparison of phasiRNA prediction results with different software

(A) Comparison results of *PHAS* gene predicted by different software based on reference transcriptome.

(B) Comparison of *PHAS* gene prediction results based on different software, PhasiHunter uses a combination of reference transcriptome and genome for phasiRNA prediction, while other software uses only reference transcriptome for phasiRNA prediction.

(C) PhasiHunter specific phasiRNA cluster plot is shown on the left, PhasiRNAnalyzer specific phasiRNA cluster plot is shown on the right.

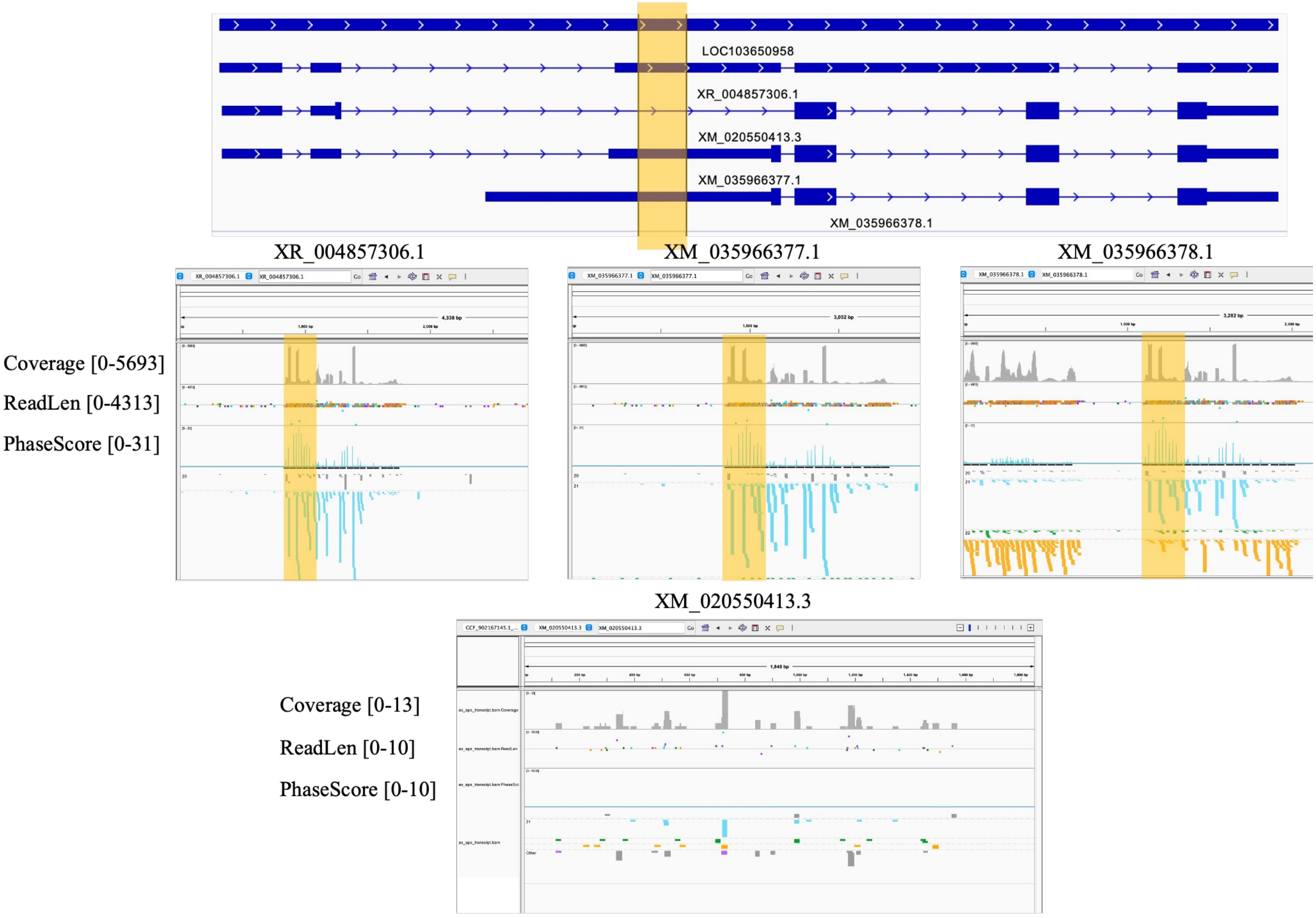

Figure S5. Alternative splicing related phasiRNAs
In this case, four transcript isoforms are produced by LOC103650958. Among these isoforms, XM_020550413.3 is unable to generate phasiRNAs due to intron clipping, whereas the other three transcript isoforms successfully generate phasiRNAs due to intron retention.

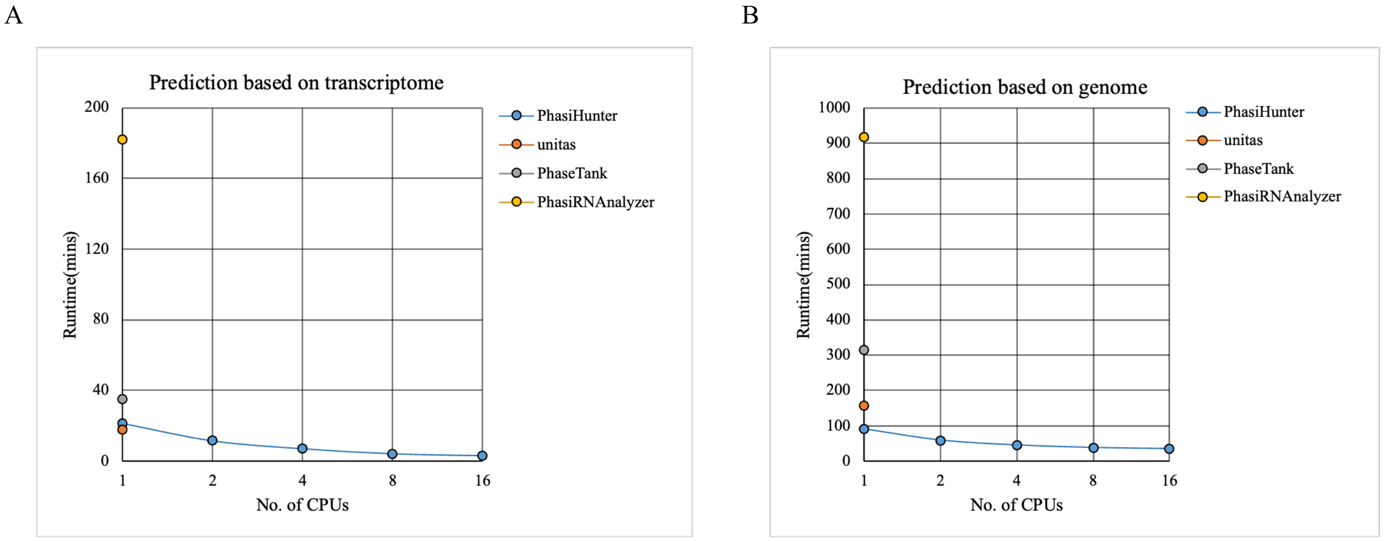

Figure S6. Comparison of phasiRNA prediction time consumption for different software
(A) The time consumption on phasiRNA prediction based on reference transcriptome by different software.
(B) The time consumption on phasiRNA prediction based on reference genome by different software.
